# Sensitivity analysis of factors in a microfluidics CFD model of coagulation and cardiac applications

**DOI:** 10.1007/s10237-025-02039-1

**Published:** 2026-01-13

**Authors:** Paolo Melidoro, Ahmed Qureshi, Steven E. Williams, Gregory Y. H. Lip, Magdalena Klis, Oleg Aslanidi, Adelaide De Vecchi

**Affiliations:** 1 School of Biomedical Engineering and Imaging Sciences, King’s College London, London, UK; 2 Centre for Cardiovascular Science, The University of Edinburgh, Edinburgh, UK; 3 Liverpool Centre for Cardiovascular Science at University of Liverpool, Liverpool John Moore’s University and Liverpool Heart & Chest Hospital, Liverpool, UK; 4 Danish Center for Health Services Research, Department of Clinical Medicine, Aalborg University, Aalborg, Denmark; 5 Cardiovascular Directorate, Guy’s and St Thomas’ NHS Foundation Trust, London, UK

**Keywords:** Thrombus formation, Coagulation, Microfluidics, Left atrium, Computational fluid dynamics

## Abstract

Coagulation is essential for haemostasis but can lead to harmful thrombus formation in conditions such as atrial fibrillation. Computational fluid dynamics (CFD) models that incorporate coagulation with blood flow can simulate this process, but their complexity often limits their use in clinical settings. This study focuses on fibrin formation during the peak thrombin phase, a brief but critical period in the thrombogram, and employs Gaussian Process Emulators to improve computational efficiency. A simplified coagulation model is integrated into a CFD framework and validated using data from an ex vivo experiment. Model inputs are varied within physiological ranges to train an emulator that predicts fibrin concentration and haemodynamic changes associated with thrombus development. A global sensitivity analysis (GSA) is performed to identify the relative influence of each input parameter. The model is then applied to a two-dimensional idealised representation of the left atrium (LA) to evaluate its suitability for cardiac simulations and to compare thrombus formation dynamics between small vessel and atrial flow. The model accurately captures fibrin formation in microchannels and the GSA and reveals potential mechanisms underlying thrombus growth in vessels while the LA simulation simulated various stages of thrombogenesis in the LA. The use of emulators enables efficient and precise predictions, enhancing the clinical feasibility of thrombosis modelling. These findings provide a foundation for the development of predictive tools to assess thrombus formation and stroke risk in patients.

## Introduction

Coagulation is a rapid and crucial response to endothelial damage, where blood forms a clot or thrombus composed of coagulation proteins, fibrin, and platelets to prevent excessive blood loss (Akbulut et al. 2023; Palta et al. 2014). While this process is essential for haemostasis, it can also lead to the formation of unwanted thrombi, both in blood vessels and the heart (Akbulut et al. 2023; Santos-Gallego et al. 2010). Conditions associated with blood stasis like deep vein thrombosis (DVT) and thromboembolism in patients with atrial fibrillation (AF) can also result in dangerous clot formation, leading to life-threatening complications (Asirvatham and van 2019; Hunt 2008; Raskob et al. 2014).

Coagulation is a complex process that involves 13 primary factors and a series of intricate biochemical reactions (Chaudhry et al. 2025). To better understand the contribution of each factor and reaction, mathematical models of coagulation have been developed (Ataullakhanov et al. 2002; Owen et al. 2024). These models provide valuable insights, potentially leading to more personalised treatment strategies and stratification for patients at risk of thrombus formation, improving outcomes and reducing complications. Due to blood stasis playing a pivotal role in thrombus formation, these mathematical models have been integrated into computational fluid dynamics (CFD) simulations to simultaneously model coagulation, blood flow, and the interplay between the 2 (Bouchnita et al. 2020; Govindarajan et al. 2016; Jimoh-Taiwo et al. 2022).

AF is the most common cardiac arrhythmia worldwide and a major risk factor for ischaemic stroke, largely due to thrombus formation in the left atrial appendage (LAA), a small, trabeculated pouch extending from the left atrium (LA) that is particularly susceptible to blood stasis during AF (Bäck et al. 2023; Melidoro et al. 2024). This clinical challenge has motivated substantial research into the use of CFD to simulate blood flow in anatomically accurate models of the LA, with the goal of identifying regions prone to stasis and assessing individual thrombotic risk (Guerrero-Hurtado et al. 2023; Mill et al. 2021; Pons et al. 2022; Zingaro et al. 2024). Image-based CFD modelling has demonstrated that haemodynamic metrics such as residence time and shear rate correlate with thrombus risk and stroke incidence (Dueñas-Pamplona et al. 2021), highlighting its potential as a mechanistic tool to enhance stroke risk stratification.

However, current clinical risk assessment schemes used to guide anticoagulant therapy, such as CHA_2_DS_2_VASc, typically do not account for mechanistic factors like blood flow dynamics or stasis, presenting an opportunity to incorporate CFD-derived metrics of LA haemodynamics (Lip and Halperin 2010; Lip and Shantsila 2009). Some recent efforts have also coupled coagulation cascade modelling with blood flow simulations in the LA to better predict thrombus formation. However, these models often lack validation against clinical or experimental data, particularly in the context of AF-related thrombogenesis in the LAA (Qureshi et al. 2025). Furthermore, the inherent complexity of the coagulation cascade makes it difficult to identify which factors most strongly influence thrombus formation. A sensitivity analysis of a validated coagulation model could therefore help identify key drivers of coagulation, enabling model simplification and improving interpretability. This underscores the need for coagulation models that are both empirically validated and systematically evaluated for input sensitivity to support reliable clinical translation in AF-related stroke risk assessment.

Using CFD to model coagulation typically involves representing the coagulation factors as scalar or species transport quantities, with the interactions between these factors modelled through source terms. In a study by Govindarajan et al., an in-vitro microfluidics coagulation experiment was successfully reproduced using a CFD model that included over 50 reaction terms and a flow time of 430 s, highlighting the potential of such models (Govindarajan et al. 2016). However, the complexity of these models comes with a significant computational cost, even at the microscale. Consequently, scaling these models to the size of the heart may be impractical due to the extensive computational resources required.

Methodological simplifications could offer a practical approach to modelling coagulation on the scale of the heart. This includes reducing the amount of transport quantities and focusing modelling on the final stage of coagulation, where thrombin breaks fibrinogen down into soluble fibrin monomers which then polymerise to form an insoluble fibrin mesh (Qureshi et al. 2023; Smine et al. 2024). As thrombin generation can take more than ten minutes to reach its peak, modelling coagulation from the point of peak thrombin can drastically reduce the required computing time. This approach is supported by the fact that assessing the coagulation response to peak levels of thrombin is already done clinically in thrombin time tests, where high levels of thrombin are added to anticoagulated patients’ blood samples and the time the blood takes to form a clot is measured where the desired range is regarded to be between 12 and 19 s (Undas 2017). Similarly, modelling coagulation from the point of peak thrombin can replicate this clinical approach by using thrombin concentrations equivalent to those observed at the peak of the thrombogram. As thrombin at peak levels has been identified as an independent risk factor for clot formation and stroke, incorporating this parameter into computational fluid dynamics models could improve their predictive accuracy. This strategy allows for more efficient simulations by avoiding the need to model the entire thrombin generation curve, thereby reducing both computational cost and complexity.

Although these assumptions make modelling coagulation in realistic anatomies more feasible, practical challenges remain when translating these models into clinical practice (Venkatesh et al. 2022). Performing these CFD simulations requires specialised expertise in CFD, a skill that is unlikely to be available in most clinical settings. Additionally, the computational resources needed to execute these simulations are typically beyond the scope of what is available in clinical environments. By utilising Bayesian statistics, Gaussian Process Emulators (GPEs) offer a promising approach to resolving these issues. A GPE is the posterior Gaussian process obtained by conditioning a Gaussian‑process prior on observed simulator runs, which provides an exact match where data exists and probabilistic prediction (with uncertainty quantification) elsewhere (Qian et al. 2025). GPEs can therefore be trained on the inputs and outputs of CFD models to be able to predict the outputs corresponding to any location in the input parameter space at a fraction of the computational expense of CFD models (Coveney et al. 2022; Rasmussen 2004; Tagade et al. 2013). GPEs can also be utilised to conduct global sensitivity analyses typically unfeasible using CFD models and assess the relative significance of each input parameter on the model’s output (Marrel et al. 2009). This approach enhances the interpretability of the model, an essential factor in ensuring that statistical models used in healthcare are both transparent and understandable (Ogbomo-Harmitt et al. 2023).

This study aims to provide proof of concept of the viability of this approach. Firstly, we will calibrate and validate a simplified model of coagulation in a vessel using results from an ex vivo coagulation experiment (Colace et al. 2012). Secondly, by varying the model inputs within physiological ranges, we will train a GPE to assess whether it can accurately predict fibrin concentration and haemodynamic changes due to the presence of a thrombus. Thirdly, the GPE will then be utilised to perform a Global Sensitivity Analysis (GSA) to identify the relative importance of each input. The model will be finally applied to a 2D idealised representation of the LA to evaluate its relevance for cardiac applications. Furthermore, we will compare the mechanisms of thrombus formation in a channel on a microscale with those in the LA to explore potential differences in thrombus dynamics.

## Methodology

**Fig. 1:**
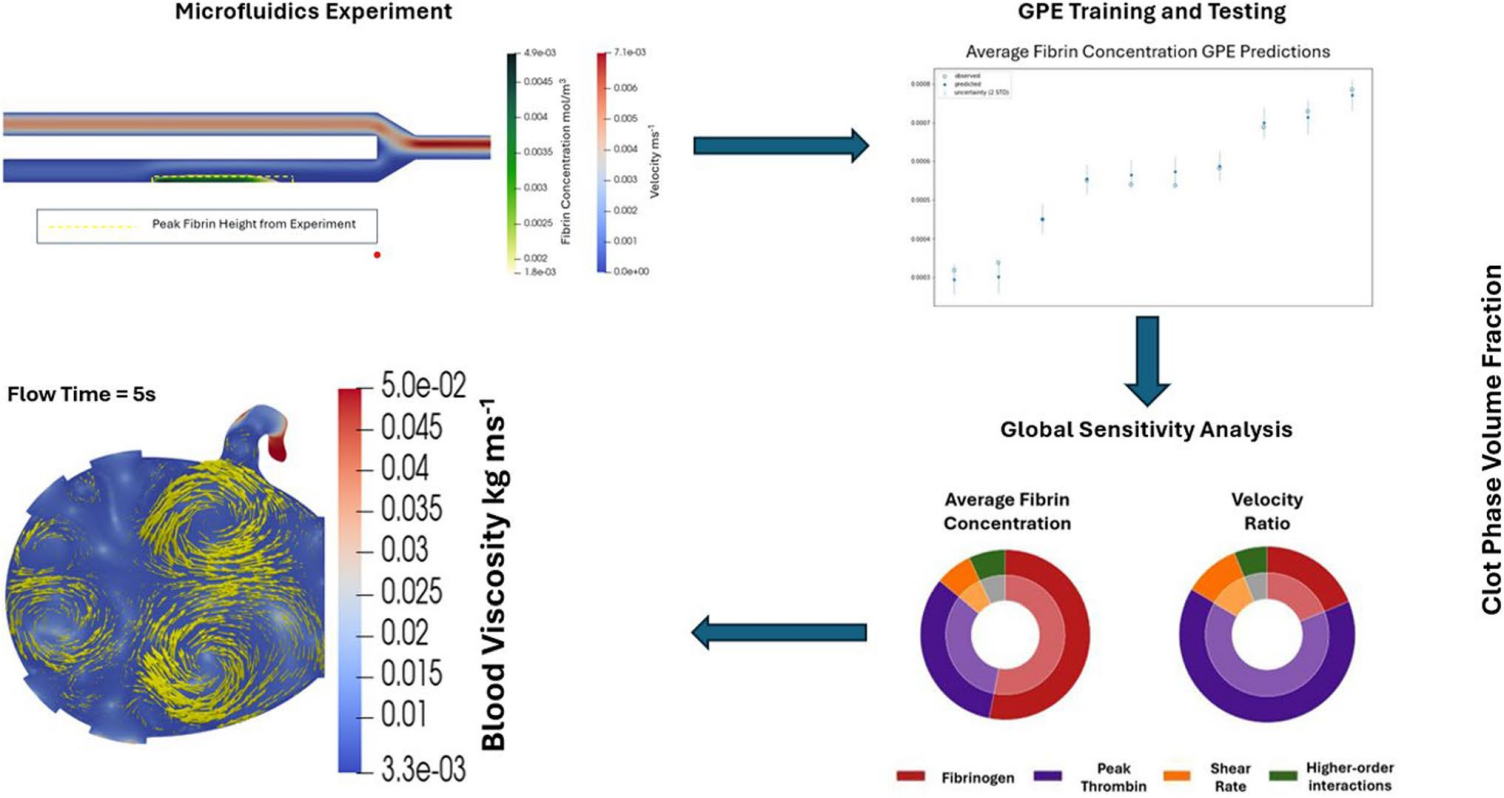
Outline of study methodology. Development of the CFD model, comparison to the ex vivo experiment for validation, generation of synthetic data to be used as inputs for the model using Latin Hypercube Sampling (LHS), use of the outputs of the model to train the GPE, use of the GPE to conduct a GSA, and application of the coagulation model to a 2D LA

The workflow of this study is outlined in Fig. 1.

### CFD model development

**Fig. 2:**
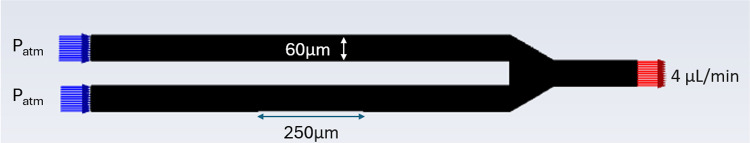
Fluid domain replicating a real experimental microfluidics apparatus, used for running the simulations with boundary conditions. The thrombotic region on the wall is indicated by the arrow

The 2D microchannel domain was created to replicate the experimental apparatus used in the ex vivo experiment conducted by (Colace et al. 2012). The inlets and the outlet of the domain were both 60 µm long while the thrombogenic surface was 250 µm as shown in Fig. 2. A mesh with a base size of 2 μm was generated using the prime mesh algorithm from ANSYS Mechanical to create a rectangular-dominant mesh with 41,301 elements. Boundary conditions were prescribed as shown in Fig. 2, i.e. a mass flow outlet with a mass flow rate of 4 µl per minute, pressure inlets with atmospheric pressure, and a no-slip condition on the wall to match the conditions of the experiment. This modelling set-up led to an initial wall shear rate (WSR) of 194 s^−1^.

The CFD simulation was performed using the finite volume solver, ANSYS Fluent. The PISO algorithm was used, enabling a relatively large time step of 0.01 s. Both the time-step and the least squares cell-based methods were employed to compute gradients, while the PRESTO! scheme was used for pressure–velocity coupling. Blood was approximated as a Newtonian fluid with a density of 1060 kg/m^3^ and a dynamic viscosity of 1060 kg/(ms). The momentum equation and all scalar transport equations were discretised using the first-order upwind scheme to maintain stability in the solution. Under-relaxation factors were set as: 0.2 for density, 0.5 for momentum, 0.5 for the scalar transport equation of thrombin, and 1 for both fibrinogen and fibrin scalar transport equations. Each simulation ran for 5 s of flow time. This flow time was chosen for computational efficiency, since the growth of thrombus was observed to plateau after 5 s in the model.

### Initial coagulation model

A simplified coagulation model was used, utilising experimentally derived biophysical equations focusing on three key coagulation components: thrombin, fibrinogen, and fibrin (Ataullakhanov et al. 2002). In the model, the initial thrombin concentration at the injury site was set to its peak physiological level. This approach allows the omission of the full temporal dynamics of thrombin generation, which typically unfold over several minutes. Since thrombin remains at its peak for approximately one minute, modelling the rise and fall of its concentration is unnecessary when simulating coagulation processes on the scale of seconds. Unlike the experiment we are using for validation, platelets were not included in this model, as the equations used were derived from experiments conducted on platelet-depleted blood (Ataullakhanov et al. 2002). This simplification would also make the model applicable to simulate clot formation in the LA, which is primarily red thrombi composed of fibrin and red blood cells.

Three scalar transport equations (for thrombin, fibrinogen, and fibrin) were coupled to the incompressible Navier–Stokes equations. As shown in Eqs. 1–4, this accounts for the convection, diffusion, and biochemical reactions of each of these proteins except for fibrin, where convection was assumed to be negligible due to the creation of an insoluble mesh. The biochemical reaction terms were implemented as source terms using user-defined functions in ANSYS Fluent. The equations, coefficients, and diffusivities used for the coagulation models were taken from previous studies (Ataullakhanov et al. 2002; Qureshi et al. 2025).

1 $$ \frac{{\partial {\mathrm{Th}}}}{\partial t} = D_{{{\mathrm{Th}}}} \Delta {\mathrm{Th}} - u\cdot\nabla {\mathrm{Th}} + R_{{{\mathrm{Th}}}} $$

2 $$ R_{{{\mathrm{Th}}}} = K_{1} \left( {1 + K_{2} {\mathrm{Th}}} \right)\left[ {K_{4} {\mathrm{Th}}\left( {1 + K_{5} {\mathrm{Th}}} \right)} \right] X \left( {1 - \frac{{{\mathrm{Th}}}}{{{\mathrm{Th}}_{{{\mathrm{Peak}}}} }}} \right) - K_{6} {\mathrm{Th}} $$

3 $$ \frac{{\partial {\mathrm{Fg}}}}{\partial t} = D_{{{\mathrm{Fg}}}} \Delta {\mathrm{Fg}} - u\cdot\nabla {\mathrm{Fg}} - K_{{{\mathrm{eff}}}} {\text{ Fg Th}} $$

4 $$ \frac{{\partial {\mathrm{Fn}}}}{\partial t} = D_{{{\mathrm{Fn}}}} \Delta {\mathrm{Fn}} + K_{{{\mathrm{eff}}}} {\text{Fg Th}} $$

where Th represents thrombin concentration, Fn fibrin concentration, *R* the reaction source term, *u* blood velocity, and Th_peak_ the peak thrombin.

To model the effect of the hydraulic resistance of fibrin on blood flow, an additional momentum source term, known as the Brinkman term, was added to the Navier–Stokes equations:

5 $$ \rho \left( {\frac{\partial u}{{\partial t}} + u\cdot\nabla u} \right) = - \nabla p + \mu \nabla^{2} u - \frac{\mu }{{K_{f} }}u $$

where* K*_*f*_ is the permeability of the fibrin mesh approximated by scaling experimental values of fibrin clots by the ratio of fibrin to the initial concentration of fibrinogen (Kuczaj et al. 2024).

The initial conditions for the biochemical concentrations were taken from the validated model by Govindarajan et al. and were set to 4.5 × 10^–3^ mol/m^3^ for fibrinogen throughout the entire fluid domain and 1.4 × 10^–5^ mol/m^3^ for thrombin, representing the peak value of thrombin, on the thrombogenic surface which was created on a portion of the fluid domain wall (Govindarajan et al. 2016).

### Gaussian Process Emulator training and testing

A LHS design was used to generate training points in the input parameters space, which included three variables, i.e. fibrinogen concentration, peak thrombin concentration, and wall shear rate (WSR), which was varied by adjusting the mass flow rate at the inlets. LHS is a statistical method that efficiently samples input variables across their entire range, ensuring broad and representative coverage of possible physiological conditions without requiring an excessive number of simulations (Karabelas et al. 2022; Qian et al. 2025). The value ranges were selected based on physiological data, with the WSR range reflecting typical shear rates observed under venous conditions. The ranges and corresponding data sources are summarised in Table 1.

**Table 1:** Range of values used for LHS with references

Input parameter	Range of values	References
Fibrinogen	0.0058–0.01176 mol/m^3^	Gaffney and Wong (2018)
Peak thrombin	1.1 × 10^–4^–3.26 × 10^–4^ mol/m^3^	Rios et al. (2022)
WSR	10–200 s^−1^	Elizondo and Fogelson (2016)

The size of the training set was determined using the empirical rule proposed by Karabelas et al., which suggests using 10 × *D* data points, where *D* represents the number of input parameters (Karabelas et al. 2022). Based on this rule, the GPE was trained using 30 data points. The GPE hyperparameters were optimised jointly by minimising the negative log-marginal likelihood of the model (Karabelas et al. 2022; Rasmussen 2004). This process was carried out using the GPErks emulation tool which leverages the GPyTorch library and the ADAM optimiser (Karabelas et al. 2022; Kingma and Ba 2017; stelong 2021). In addition to the 30 training points, 10 testing points were created using a separate LHS dataset. The GPE was trained to map input parameters to two output features: the average fibrin concentration across the fluid domain and the ratio of flow exiting through the bottom channel to the total flow exiting the apparatus, both measured at the final time step, with the latter quantifying the occlusive effect of the clot. The accuracy of each GPE was assessed using the *R*^2^ score and the individual standard error (ISE) score. The median ISE indicates the percentage of GPE predictions that lie within two standard deviations of the observed value.

### Global sensitivity analysis

A GSA was conducted to quantify the relative contributions of the three input parameters described above to the variance in the model outputs. The Sobol indices were derived by decomposing the total output variance into the individual variances linked to each input, as well as their interactions (Marrel et al. 2009). The Saltelli method was employed for the GSA (Longobardi et al. 2020). The total number of model evaluations required to estimate the sensitivity indices was calculated using the formula *N* × (2 × *D* + 2) (Saltelli et al. 2010), where *N* is the desired number of Monte Carlo samples and *D* is the number of input parameters. For this analysis, *N* was set to 1024 Monte Carlo samples, and* D* was 3, resulting in 9216 model evaluations per GPE. To account for emulator uncertainty, the sensitivity indices were predicted 1000 times, yielding an index distribution rather than single values. The final sensitivity indices were then obtained as the mean of these distributions.

### Application in a 2D left atrium

The coagulation model previously applied in a 2D microchannel was extended to an idealised 2D model of the LA. This model was constructed using a synthetic representation of the LAA designed to replicate the commonly observed “chicken wing” morphology described in clinical studies (Di Biase et al. 2012). Geometric parameters for the main LA cavity were obtained from published data (Abdelmoneim and Mulvagh 2014; Qureshi et al. 2020). The complete 2D geometry was developed using Shapr3D (Siemens).

**Fig. 3:**
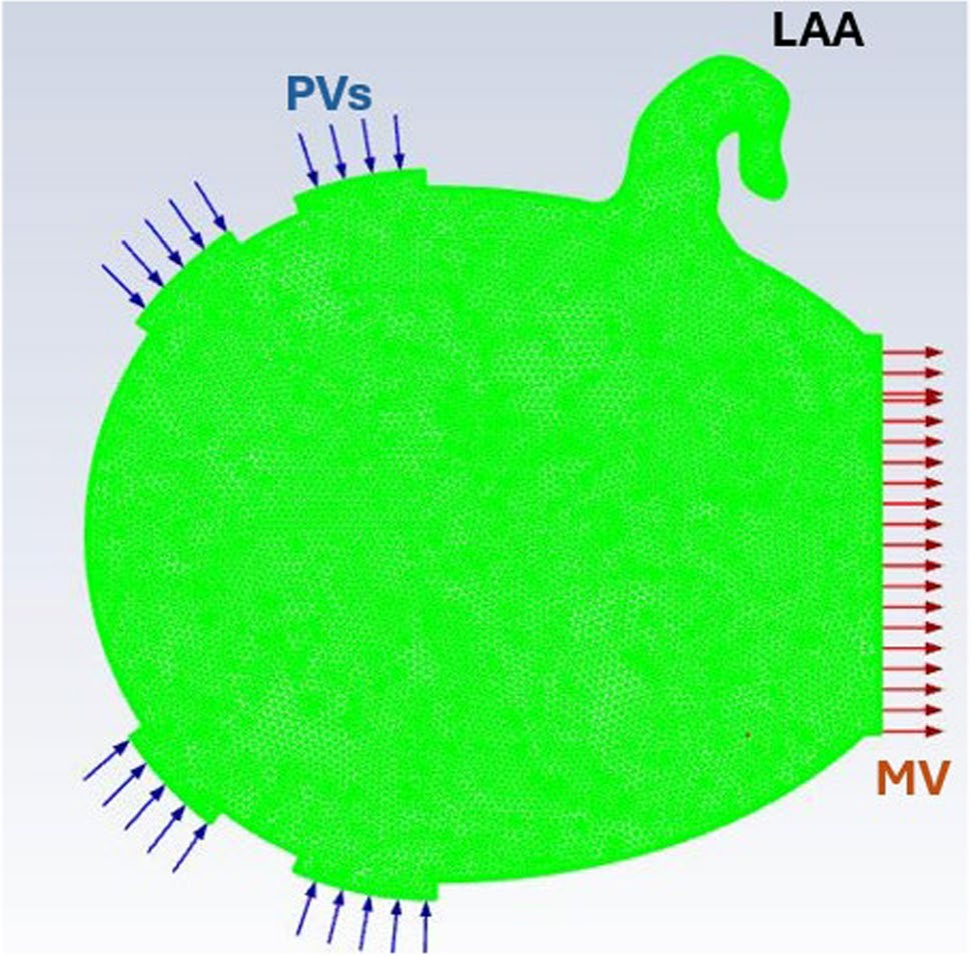
2D Idealised left atrium with boundary conditions

The 2D LA mesh consisted of 22,608 elements, including three boundary layers to ensure accurate resolution of near-wall flow where clot initiation occurred. The remainder of the mesh was composed of triangular elements. The 2D LA model with the corresponding boundary conditions is shown in Fig. 3.

To improve numerical stability and reduce computational time, fibrin gelation was represented using a viscosity hill function, following the approach of Qureshi et al., where viscosity increases with fibrin concentration (Qureshi et al. 2025). This approach was used instead of incorporating a momentum term to model permeability, as was done in the microchannel simulations.

A multiphase Volume of Fluid (VOF) model was employed to capture both the non-Newtonian behaviour of blood and the evolving viscosity of the forming clot. In this model, blood and clot were treated as separate phases, allowing different viscosity functions to be applied to each. The non-Newtonian model assumed an average fibrinogen concentration of 3 g/L and a haematocrit of 0.45, consistent with the model used by Melidoro et al. for simulations of non-Newtonian blood flow in the LA (Melidoro et al. 2024).

**Fig. 4:**
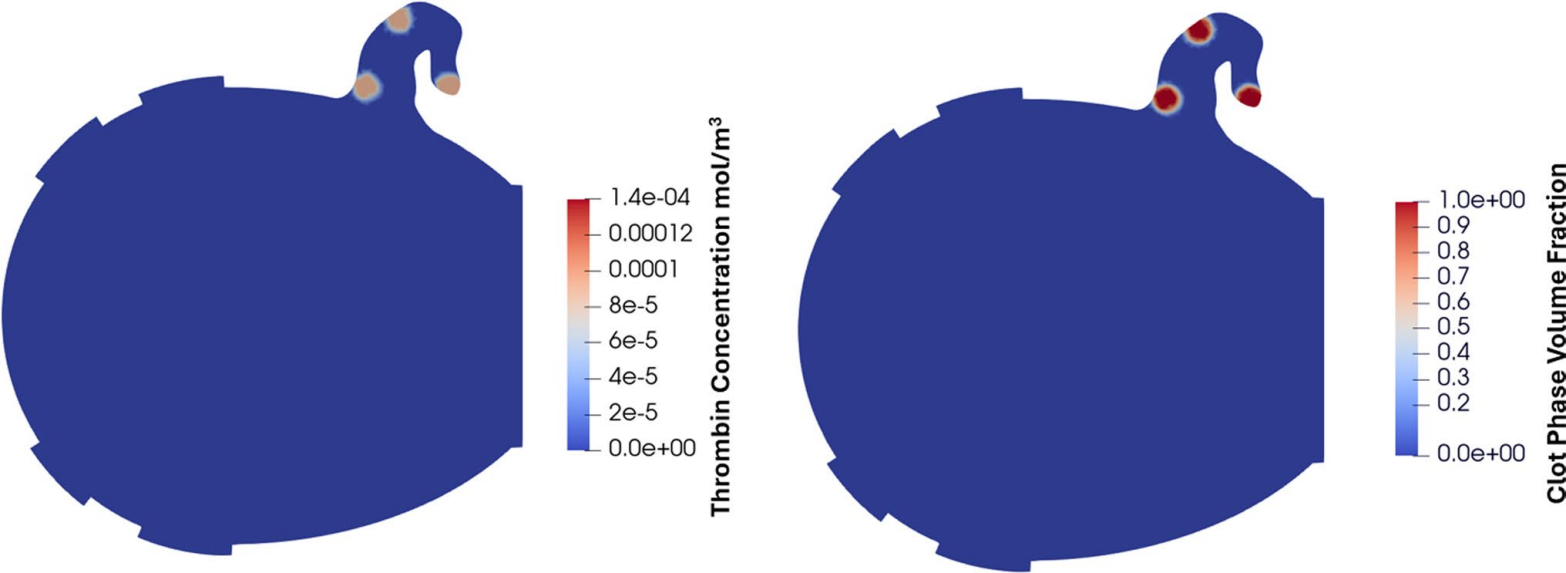
Initialisation of thrombin generation and clot phase in the left atrial appendage (LAA). Thrombin generation and the clot phase were initiated at the orifice, neck, and tip of the LAA, representing regions prone to thrombus formation

The same solver settings were used as in the microchannel simulations. Thrombin generation was initiated with a concentration of 9 × 10^−5^ mmol/L at three locations within the LAA: the orifice, the neck, and the tip, corresponding to regions where thrombus formation has been reported. This approach to initialising thrombin generation is in line with previous approaches to thrombus modelling in the LA (Qureshi et al. 2025). The volume fraction of the clot phase was initiated as 1 in the same regions where thrombin was initiated and the volume fraction was initiated at 0 for the rest of LA, as shown in Fig. 4. Flow at the PVs was imposed using a sinusoidal velocity profile with a peak magnitude of 0.4 m/s, while the mitral valve was defined as an open outlet. This simulation was run for 10 s of flow time due to both fibrin concentration and the spatial growth of fibrin both plateauing between 5 and 10 s flow time.

## Results

### Comparing model with ex vivo experiment

**Fig. 5:**
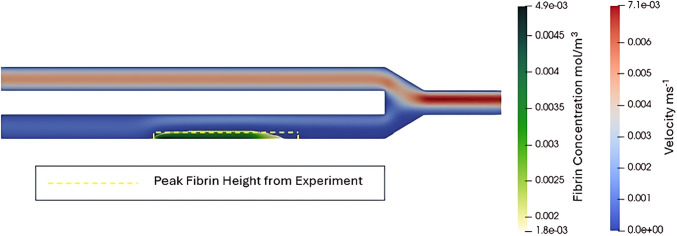
Comparing the height of the fibrin clot from the CFD model to the height of the fibrin deposit from the experiment

Figure 5 shows how the fibrin clot formed in the initial CFD model after 5 s compares to what was observed experimentally by (Colace et al. 2012). The velocity and fibrin concentration observed in the CFD model are shown as well as the peak height of the fibrin deposition observed experimentally. Figure 5 shows the fibrin distribution from the CFD model, highlighting regions where fibrin concentration exceeded 20% of the initial fibrinogen concentration, corresponding to the gel point in fibrin polymerisation, where clot formation begins as fibrinogen is incorporated into the gel (Weisel and Litvinov 2013, 2017). The simulated fibrin clot height closely matched experimental observations at the same time point (25 vs 21.3 µm), with a 14.8% error.

**Fig. 6:**
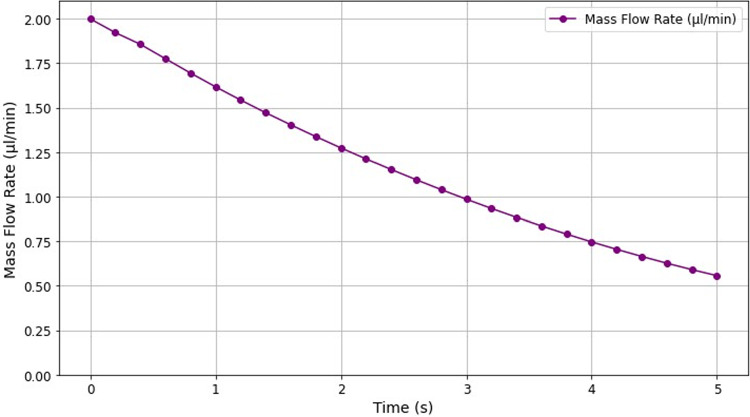
Mass flow rate of the bottom channel plotted over time. As the clot grows throughout the simulation, it gradually occludes the bottom channel, leading to a gradual reduction in the mass flow rate

Figure 6 illustrates the reduction in mass flow rate through the bottom channel as the blood clot grew over time. Initially, the mass flow rate was 2 µl/min, decreasing to 0.56 µl/min after 5 s (72% reduction). This result differed from experimental observations, which showed near-total occlusion of the flow. However, this 28% difference in the occlusiveness can be explained by the presence of platelets in the experiment, which played a significant role in the occlusion, contributing substantially to the reduction in flow within the channel (Colace et al. 2012).

### Gaussian Process Emulator

**Fig. 7:**
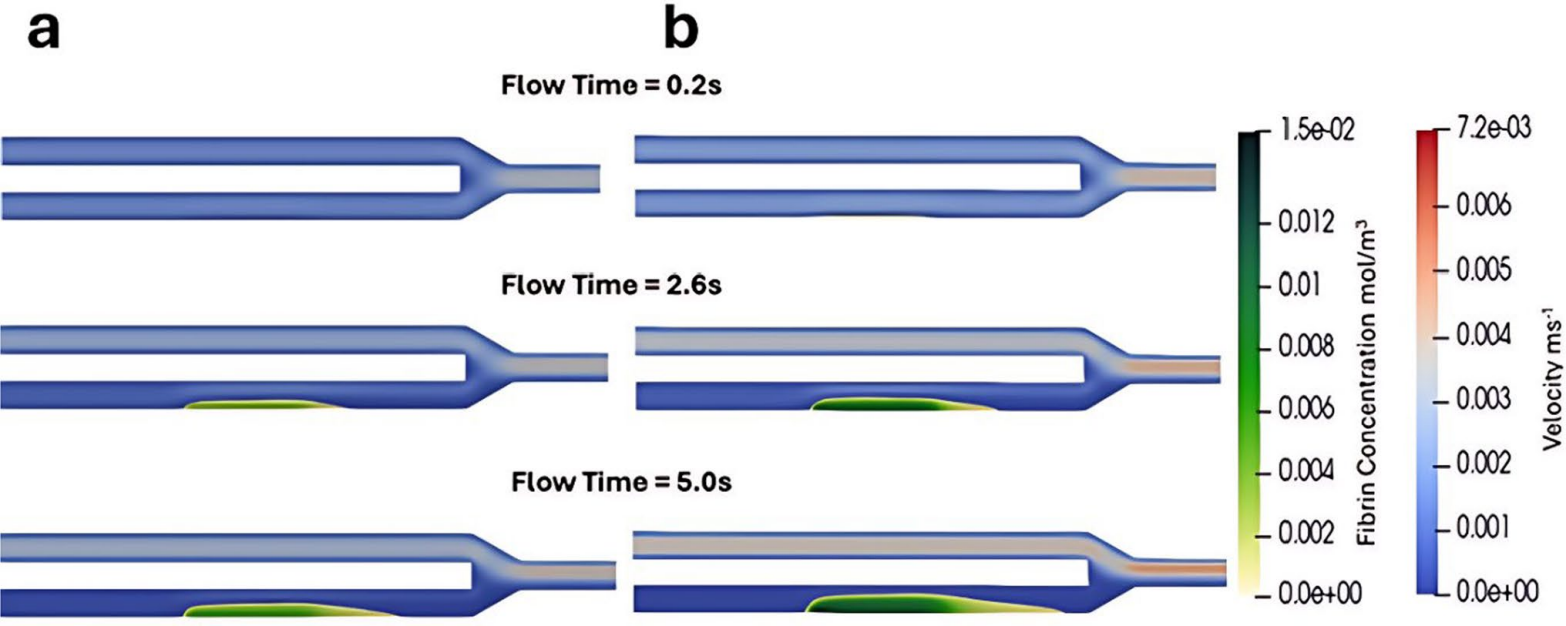
Time evolution of fibrin concentration and blood flow velocity in the cases with the lowest (**a**) and highest (**b**) average fibrin concentration

A range of values representing normal physiological conditions generated by LHS were used as inputs for the CFD model, as outlined in Table 1. The two cases with the highest (8.28 × 10⁻^4^ mol/m^3^) and lowest (3.4 × 10⁻^4^ mol/m^3^) fibrin concentrations at the end of the simulation (averaged spatially across the entire fluid domain) are shown in Fig. 7, where fibrin concentration and blood are presented at three time points: the start (0.2 s), middle (2.6 s), and end (5 s) of the simulation.

In the lowest concentration case (Fig. 7a), the inputs were fibrinogen 7.94 × 10⁻^3^ mol/m^3^, peak thrombin 1.1 × 10⁻^4^ mol/m^3^, and WSR 119 s⁻^1^. In the highest concentration case (Fig. 7b), the input parameters were fibrinogen 1.12 × 10⁻^2^ mol/m^3^, peak thrombin 3.08 × 10⁻^4^ mol/m^3^, and shear rate 142 s⁻^1^. Figure 7 demonstrates how significantly both the spatial growth of the blood clot and the fibrin concentration can vary, even within normal physiological conditions. The average fibrin concentration measured at the simulation’s conclusion was 3.51 × 10^–4^ mol/m^3^ in the lowest concentration case and 7.85 × 10^–4^ mol/m^3^ in the highest concentration case, while the velocity ratio varied from 0.0356 to 0.0994.

**Fig. 8:**
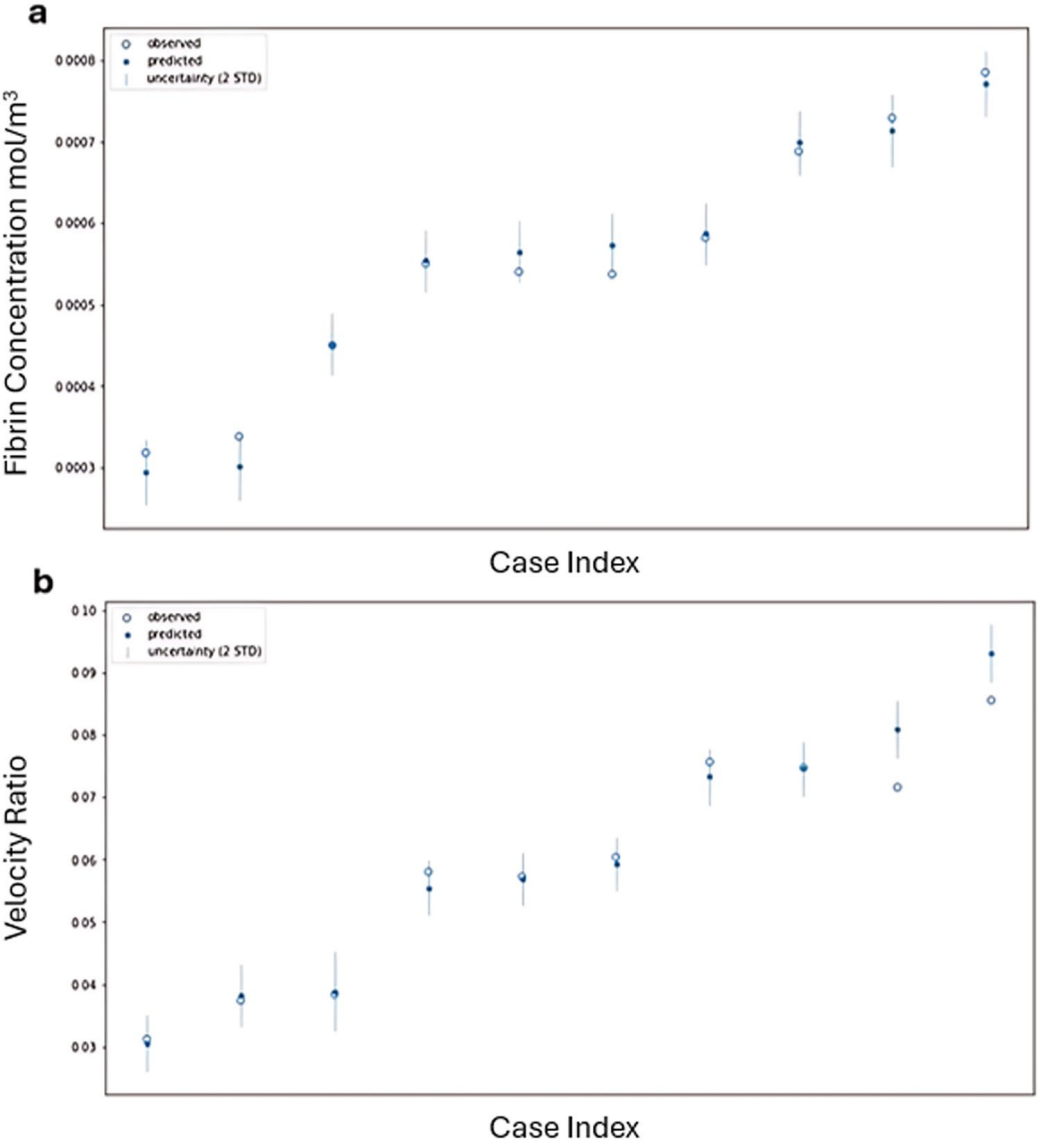
GPE predictions for both average fibrin concentration (**a**) and velocity ratio (**b**) compared to the real values from the CFD model

The outputs of the 30 simulations using the LHS training dataset were employed to train the GPE. Figure 8a and b illustrates the accuracy of the GPE’s predictions for the testing dataset, showing whether the GPE predictions fell within 2 standard deviations of the corresponding CFD model outputs. In Fig. 8a, all 10 GPE predictions for average fibrin concentration were within 2 standard deviations of the CFD model outputs, yielding an ISE of 100. Figure 8b shows that 8 of the 10 GPE predictions for the velocity ratio were within 2 standard deviations of the CFD outputs, resulting in an ISE of 80. Additionally, the *R*^2^ values for the GPE’s predictions were 0.9794 for average fibrin concentration and 0.9477 for the velocity ratio, indicating that the GPE achieved a high level of accuracy in predicting both outputs.

### Global sensitivity analysis

**Fig. 9:**
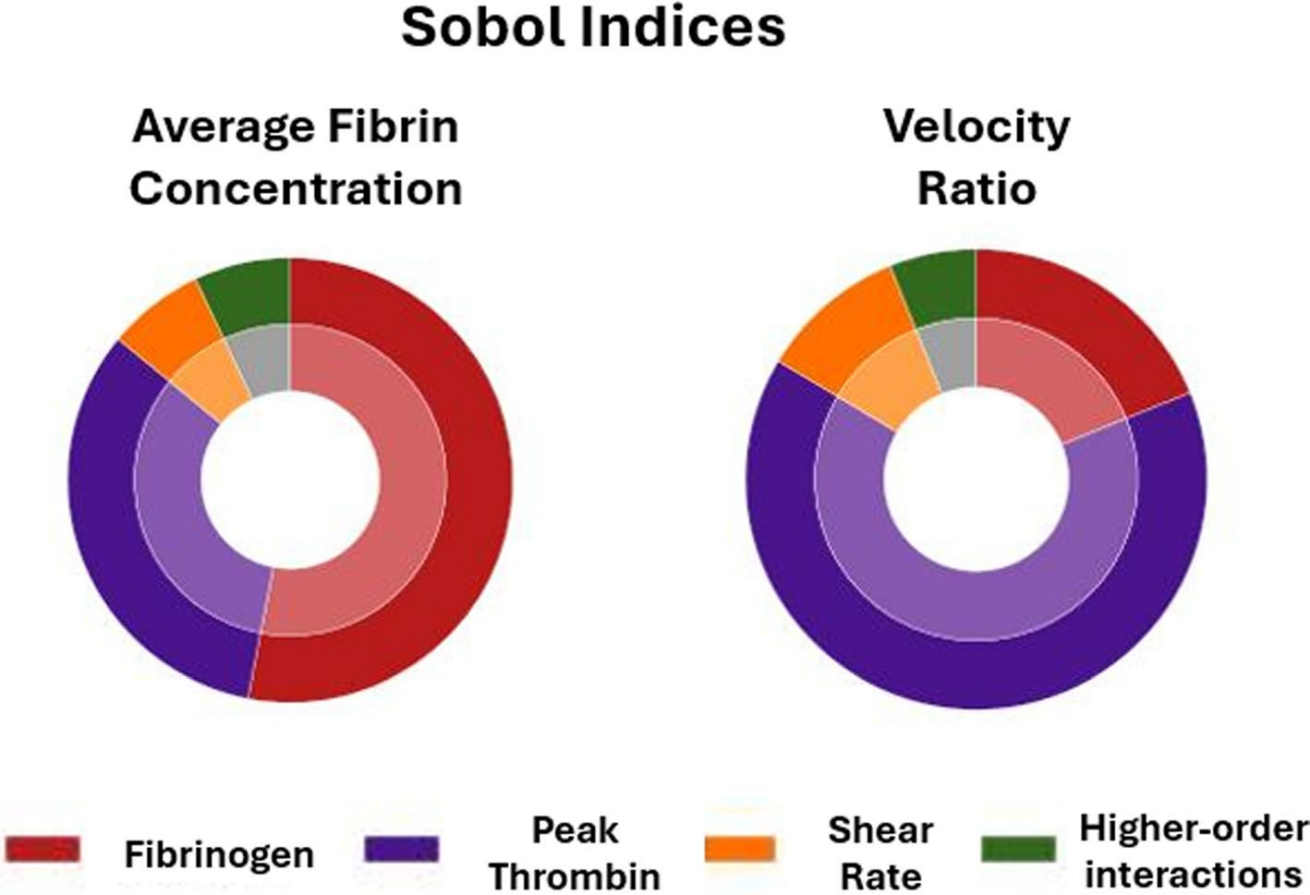
Sobol indices for each input parameter, quantifying the influence each input has on both outputs, average fibrin concentration, and velocity ratio

The trained GPEs were used to conduct a GSA to quantify the influence each input parameter had on both outputs by finding the Sobol indices of each input parameter. Figure 9 illustrates the significance each input parameter had on both average fibrin concentration and the velocity ratio. Fibrinogen had the largest impact on the average fibrin concentration while thrombin had the largest impact on the velocity ratio. While the shear rate did influence both outputs, it was relatively small compared to fibrinogen and thrombin.

### 2D left atrium simulation

**Fig. 10:**
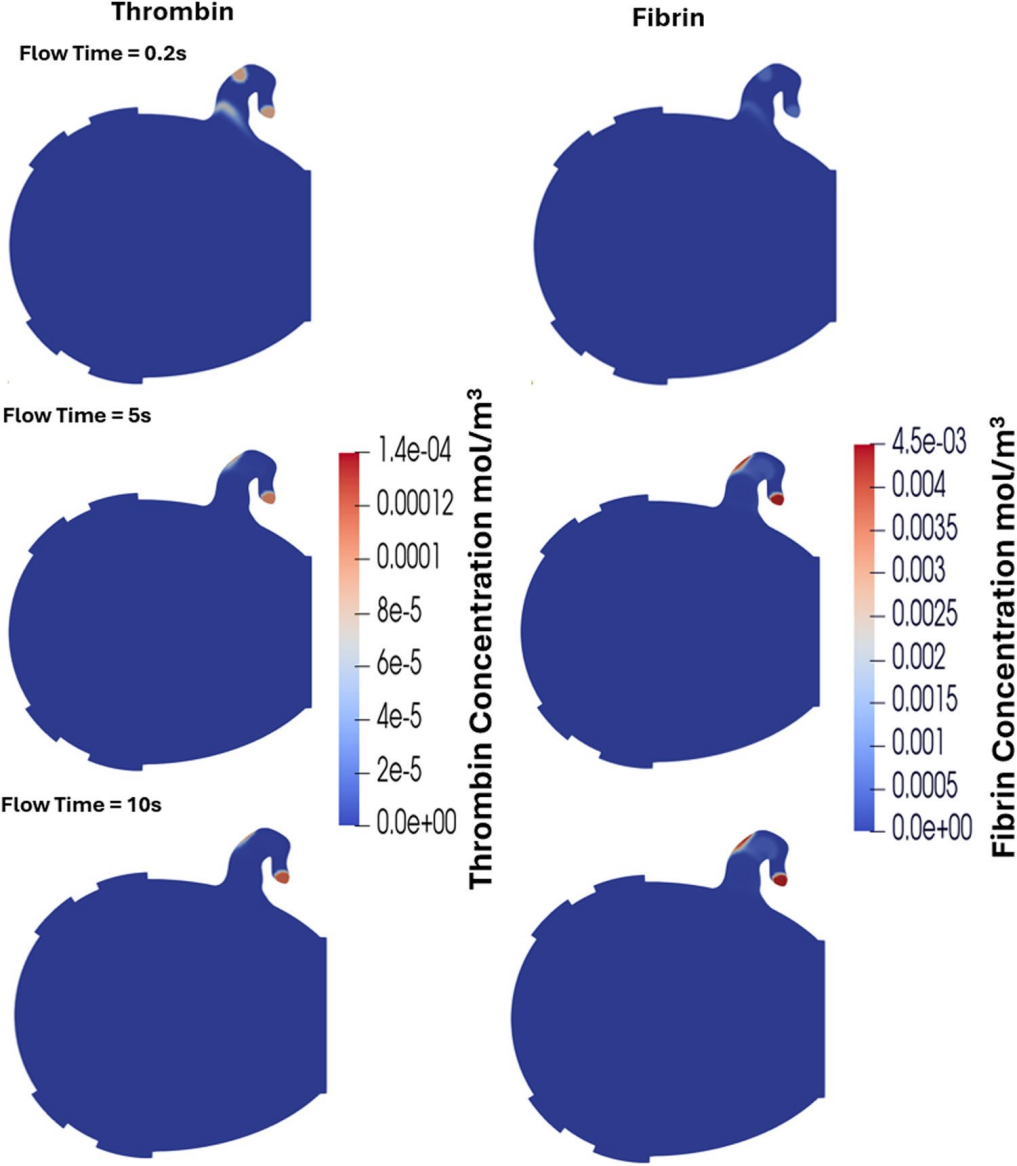
Fibrin and thrombin concentrations in the LA at the start, middle, and end of the simulation. The gradual increase in thrombin and fibrin concentration as well as their spatial growth throughout the simulation can be observed

Figure 10 shows fibrin concentration in the 2D LA at three time points: 0.2 s (start), 5 s (midpoint), and 10 s (end) of the simulation. Throughout the simulation, both thrombin and fibrin gradually increased in the LAA at the neck and tip before plateauing, while at the orifice they were washed away early in the process. At 5 s, the fibrin concentration reached 4.5 × 10⁻^3^ mol/m^3^ at the tip and in part of the neck region where thrombin was initiated near the wall, indicating that all fibrinogen in this area was converted to fibrin. This finding suggests that blood flow in these regions was insufficient to remove thrombin and fibrin effectively. By 10 s, thrombin concentration at the tip and neck reached its peak of 1.4 × 10⁻^4^ mol/m^3^, while fibrin levels in the LAA remained similar to those observed at 5 s.

**Fig. 11:**
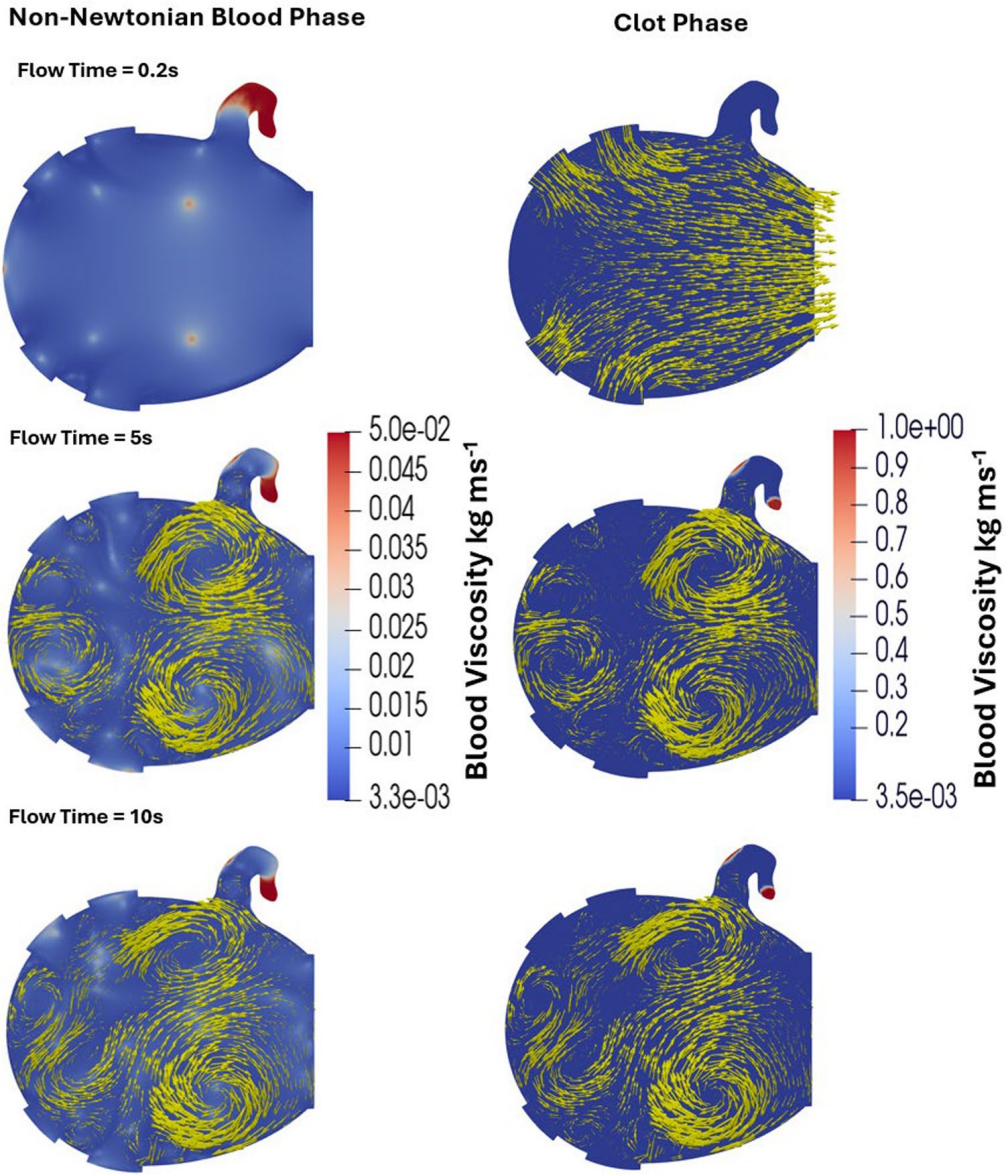
Multiphase viscosity distribution in the LA at 0.2 s, 5 s, and 10 s from the VOF model, showing the non-Newtonian blood phase and the fibrin-dependent clot phase during the thrombus gelation process. The phases are presented using different viscosity scales, as the maximum viscosity due to fibrin gelation is several orders of magnitude greater than that resulting from non-Newtonian blood effects. The yellow arrows highlight the blood flow with the vectors being scaled by velocity magnitude

Figure 11 illustrates the variation in viscosity within the LA resulting from the non-Newtonian behaviour of blood in one phase of the VOF model, as well as the fibrin-dependent viscosity changes associated with the thrombus gelation process. The non-Newtonian effects appear immediately since they depend entirely on the local shear rate. At 0.2 s, these effects are most pronounced because the washout vortex in the LAA has not yet developed. By 5 s and 10 s, when the LAA washout vortex is fully formed, the influence of non-Newtonian behaviour decreases spatially. However, elevated viscosity persists near the LAA tip, where blood stasis remains significant. For the clot phase, the fibrin-dependent viscosity increases over time, following the same temporal and spatial pattern as the fibrin concentration observed above.

## Discussion

This study is the first to perform a sensitivity analysis of the factors involved in a microfluidic channel CFD simulation to identify the main drivers of thrombogenesis and venous occlusion at shear rates representative of venous flow. It examines both haematological factors and the severity of blood stasis, providing quantitative insight into the extent to which each of these parameters influences thrombus formation in such conditions. While some studies used Gaussian processes to predict blood flow, cardiac mechanics parameters, or electrophysiological parameters, it is the first study to use GPEs to predict the outcomes of a microfluidic coagulation model, offering a foundation for future developments aimed at predicting venous thrombosis with minimal computational cost (Di Achille et al. 2018; Karabelas et al. 2022; Noè et al. 2019; Qian et al. 2025). In addition, this study is the first to employ a multiphase VOF model to simultaneously simulate blood clot formation and non-Newtonian behaviour principally driven by red blood cell (RBC) aggregation in an idealised LA. By assigning distinct phases to blood and clot, the model captures two essential aspects of LA thrombogenesis: the non-Newtonian behaviour of blood and the progressive gelation of the clot associated with increasing fibrin concentration.

In this study, we have shown that we can reproduce the growth of fibrin observed experimentally with a simplified model of coagulation that incorporates three central biochemical species in the coagulation cascade—thrombin, fibrinogen, and fibrin. Our model initiated thrombin by setting its peak value on the wall of the microchannel, replicating the dimensions of the thrombogenic surface in the experiment. By setting thrombin at its peak value, as opposed to incorporating the entirety of the thrombogram where usually thrombin peaks after 10 min, we have shown that such a simplification can vastly reduce computational time and expense and still produce accurate results in terms of fibrin concentration. It is important to note that the thrombin generation curve presented by Govindarajan et al. was not used as a predefined input, but rather emerged as the result of solving a system of ordinary differential equations (ODEs) representing the biochemical reactions involved in thrombin generation (Govindarajan et al. 2016). Personalising such a mechanistic model for individual patients would require measuring numerous biochemical species, which may not be feasible using standard blood tests. In contrast, using the peak thrombin concentration as a parameter would only require obtaining the patient’s thrombogram. This approach not only simplifies the data collection process but also ensures that the peak thrombin value is directly measured from the patient’s blood, offering a high degree of accuracy compared to model-derived estimates.

An important consideration is that the experiment from which the equations of our model were derived was conducted with platelet-free blood, while the experiment we were using as validation included platelets; this was purposely done due to the motivation of translating our model for cardiac applications, specifically the LA, where thrombi observed in this region are “red” thrombi composed primarily of fibrin and red blood cells as opposed to “white” thrombi composed of mainly platelets (Ataullakhanov et al. 2002; Lip and Shantsila 2009). Despite this differing from the experimental setup, the growth in fibrin in the model was still comparable to that of the experiment. The reduction in mass flow rate due to the occlusion of the lower channel, although high (72% reduction), was not as high as that of the experiment where there was almost total occlusion. This was likely caused by the platelets in the experiment providing an additional occlusive effect in addition to the occlusion from the fibrin gel. It should be noted, however, that in a second experiment performed by (Colace et al. 2012), when conversion of fibrinogen to fibrin was inhibited, the mass flow rate remained above half of its original value, suggesting that fibrin is primarily responsible for occlusion. This was supported by our model where fibrin generation was able to reduce the mass flow rate by 72% without the inclusion of platelets (Colace et al. 2012).

Varying the input parameters—fibrinogen and peak thrombin concentration, and shear rate—in the LHS design revealed that even within physiological ranges, these clinical parameters can significantly affect thrombus characteristics. Notably, they influence both fibrin concentration and clot occlusiveness, the latter being largely determined by the spatial growth of the thrombus. These results suggest that the chosen inputs are clinically meaningful for predicting thrombogenesis, particularly in pathologies where red thrombus formation is a concern.

The two instances where the GPE failed to predict the velocity ratio within two standard deviations corresponded to the cases with the highest initial WSR, meaning the cases where the velocity was highest. This suggests that the GPE may not have encountered sufficient training cases to effectively learn the flow behaviour in these scenarios, or that the relationship between fibrin growth and flow in these conditions is more complex, making it challenging for the GPE to model accurately. Therefore, future studies should prioritise expanding the training dataset to better cover the higher range of WSR values, ensuring the model can more accurately capture the associated flow dynamics.

The GSA, conducted on outputs taken after the 5 s of simulation time, gave particularly interesting results, specifically that fibrin concentration was primarily affected by fibrinogen concentration, while the occlusiveness and growth of the clot were primarily affected by peak thrombin concentration, demonstrating how different biochemical species can affect the properties of thrombi in different ways. Elevated levels of peak thrombin and fibrinogen concentration have both previously been linked to venous thromboembolism (VTE), where red thrombus forms due to blood stasis in veins (Lutsey et al. 2009; Verzeroli et al. 2024). It has also been shown that higher fibrinogen levels can lead to more stable clots, and clots observed experimentally in mice with high fibrinogen levels have been resistant to thrombolysis (Machlus et al. 2011; Wolberg and Sang 2022). This demonstrates a possible mechanism for why high fibrinogen is correlated with increased VTE risk. The fact that our results show fibrinogen primarily affected fibrin concentration rather than occlusiveness supports these findings, as clots with higher fibrin content are less permeable, which is known to increase clot stability and resistance to thrombolysis (Kuczaj et al. 2024). In contrast, spatial clot growth being primarily determined by peak thrombin concentration suggests a different mechanism for thrombin’s role in VTE. Increased thrombin may drive the clot to grow more rapidly towards the centre of the vessel, where higher blood flow increases the likelihood of clot embolisation.

In our global sensitivity analysis, wall shear rate (WSR) showed relatively low influence on both average fibrin concentration and velocity ratio, despite being varied across a wide physiological range from 10 to 200 s⁻^1^. While this may appear to contradict the role of blood stasis in thrombus formation as described by Virchow’s triad, it is important to note that the entire range of WSR values used in this study corresponds to conditions under which thrombosis is already known to occur. This suggests that all values are sufficiently low to trigger clot formation, and therefore, variations within this prothrombotic range may not result in significant changes in model outputs. The apparent insensitivity does not imply that WSR is unimportant, but rather that its effect plateaus once a certain threshold for thrombus initiation is crossed. Furthermore, we observed that GPE performed less accurately in high velocity ratio cases, which typically correspond to higher WSR. This may reflect limitations of the emulator or underlying model in capturing complex interactions at higher share rates such as the transport of the biochemical species and the interaction between blood flow and the porous fibrin structure.

The 2D LA simulation employed a multiphase model to simultaneously capture thrombus formation and the non-Newtonian behaviour of blood. This represents the first model to incorporate both phenomena, which is significant because the underlying mechanism of blood’s non-Newtonian behaviour, RBC aggregation, is also responsible for the imaging manifestation of spontaneous echo contrast (SEC). SEC has been identified as a stronger predictor of stroke than low LAA velocity, defined by peak velocities below 20 cm/s (González-Torrecilla et al. 2000; Melidoro et al. 2025). Furthermore, severe SEC has been characterised as an intermediate stage of coagulation that occurs after the onset of SEC due to blood stasis but before the formation of a solid thrombus. This stage indicates that coagulation has begun, although fibrin concentration has not yet reached the threshold for gelation and thrombus solidification. Therefore, it is essential to model the entire thrombotic environment, encompassing both stasis-induced SEC resulting from RBC aggregation and the subsequent transition to a mature thrombus (Melidoro et al. 2025). The proposed multiphase model achieves this by accounting for viscosity changes arising from RBC aggregation and the progressive increase in fibrin concentration.

The trained GPEs demonstrated high accuracy in predicting both outputs, which is a promising outcome for ensuring clinical viability. This is particularly important because models must operate within the constraints of typical healthcare settings, where resources are often insufficient for running computationally expensive CFD simulations. In comparison, GPEs can produce predictions on standard personal computers and do not require specialised knowledge of CFD, making them far more accessible and practical in real-world clinical environments.

While this study presents a proof-of-concept CFD experiment using a 2D idealised LA geometry to demonstrate the feasibility of applying our coagulation model in cardiac domains, future work will involve performing simulations in patient-specific 3D LA geometries derived from medical imaging. This will allow us to train GPEs capable of predicting key coagulation factors based on both the model inputs used here and realistic individual anatomical variations. We also plan to extend this approach to other cardiovascular applications, including abdominal aortic aneurysms, where CFD has been extensively used to study haemodynamic conditions associated with patient outcomes (Di Achille et al. 2014; Manta and Tzirakis 2025).

To translate this work into a clinically viable model, several key steps must be undertaken. First, real patient data must be obtained, including blood clotting profiles with fibrinogen concentrations and where possible an indication of thrombin activity to accurately model the coagulation process. Additionally, CT scans will be required to generate anatomically accurate representations of the LA, including the left atrial appendage (LAA), which are essential for creating the CFD domain. These anatomical models must then be converted into high-quality meshes suitable for CFD simulations.

To validate the predictive accuracy of the model, prospective longitudinal data from patients will also be necessary. This includes follow-up information spanning from 6 months to 5 years after the initial CT scans pertaining to whether patients have suffered a stroke, enabling comparison between CFD-derived metrics and actual clinical outcomes. Such longitudinal validation is critical to assess the model’s utility in predicting thromboembolic risk and ensuring its effectiveness in real-world clinical settings.

A limitation of this study is that simulations were initiated at the point of peak thrombin and only run for five to ten seconds, which does not capture the full duration of thrombus formation. This choice was motivated by computational efficiency, as peak thrombin is reached after around ten minutes after the initial release of tissue factor. By focusing on the period of the thrombogram where thrombin is close to its peak, we can run simulations that do not require up to tens of minutes of flow time. However, this approach omits the initiation phase of thrombin generation, during which important spatial and temporal gradients can develop and influence downstream coagulation processes. As such, while the short simulation window captures key features of coagulation, it may not fully represent localised thrombin dynamics. Future work should consider incorporating the full thrombin generation profile to better account for these early spatial effects (Duchemin et al. 2008) and to further validate the predictive capabilities of surrogate models trained on short-term outputs. Similarly, the simplified 2D LA model, while designed to enable faster simulations to explore general thrombus formation mechanisms, does not represent the complex haemodynamics involved in a real anatomy. As such, more physiologically detailed models may be necessary to achieve greater precision in simulating thrombus development.

While the model purposely did not include platelets due to the deliberate choice of using equations that were empirically derived from platelet-free blood to model red thrombus formation, the experiment which we used for validation did include platelets. Although this did not lead to a large difference in terms of thrombus growth, this did lead to a 28% discrepancy in terms of the occlusiveness of the blood clot, due to the occlusiveness of platelets playing a significant role in the total occlusiveness of the blood clot in the experiment.

While the exclusion of a direct model for the activation and aggregation of platelets is a limitation due to platelet activation initiating thrombin generation, our model focuses on the downstream coagulation cascade by treating thrombin as a direct input. This simplification may reduce the model’s ability to capture early platelet-driven events during thrombus initiation. However, since red thrombi in the LA are mainly composed of fibrin and red blood cells, and clinical evidence shows that anticoagulants like warfarin are more effective than antiplatelet agents in AF, we believe this approach captures the primary mechanisms behind thrombus propagation in these patients (Watson et al. 2009). Future work could expand the model to incorporate platelet dynamics for a more complete representation of thrombus initiation and growth.

## Conclusion

This study presents a simplified yet effective computational model of coagulation that captures the key dynamics of fibrin growth by focusing on three core biochemical species involved in coagulation, i.e. thrombin, fibrinogen, and fibrin. By initiating thrombin at its peak concentration rather than simulating the full thrombogram, we significantly reduced computational cost while maintaining accuracy in predicting fibrin formation. The GSA revealed that fibrinogen concentration primarily drives the fibrin concentration and subsequently hydraulic resistance, while peak thrombin concentration dictates the rate of clot growth. The trained GPEs proved highly accurate and computationally efficient, highlighting their potential for clinical application, where resources for running complex CFD models are usually inadequate. Furthermore, 2D simulations in the LA demonstrated physiologically realistic fibrin accumulation patterns, particularly in areas of high stasis and anatomical curvature, and offered a plausible mechanistic link between SEC, LA sludge, and thrombus formation. These insights not only validate the model but also offer a foundation for predictive tools aimed at assessing thrombus risk in cardiac settings, especially within the LAA. Overall, this work advances the understanding of fibrin clot formation and supports the development of clinically viable tools for risk assessment and therapeutic planning in cardiovascular medicine.

## Data Availability

No datasets were generated or analysed during the current study.
